# Current evidence on circRNAs as potential theranostic markers for detecting chemoresistance in breast cancer: a systematic review and meta‑analysis

**DOI:** 10.1038/s41598-022-26220-z

**Published:** 2022-12-20

**Authors:** Zixin Zhu, Hui Jiang, Jingling Xie, Xinrui Jin, Baolin Li, Jinbo Liu

**Affiliations:** Department of Laboratory Medicine, The Affiliated Hospital of Southwest Medical University, 25 Taiping Street, Luzhou, 646000 Sichuan People’s Republic of China

**Keywords:** Cancer, Breast cancer, Tumour biomarkers

## Abstract

This study assessed the value of circRNAs (circular RNAs) as prognostic markers in BC (breast cancer). We searched pertinent studies on the PubMed, Embase, and Web of Science online databases published according to PRISMA guidelines. A random-effects model for meta-analysis was used to assess the combined effect size of the HRs (hazard ratios) of the included studies. The heterogeneity test used Cochran's *Q*-test and *I*^2^ statistics. Thirty of the 520 trials retrieved were included in the systematic review. A total of 11 chemotherapeutic agents were used in the included studies. A total of 30 studies on 30 circRNAs were included in the systematic review. Of the 30 relevant circRNAs, 28 were upregulated and two were downregulated in breast cancer versus normal samples, and both were associated with increased drug resistance. Nine of 30 studies were used for the meta-analysis. The results of the meta-analysis showed that the groups with circRNA upregulation and circRNA downregulation showed the same prognostic risk (HR = 1.37, 95% Cl: 0.80–2.36, *I*^2^ = 63.7%). The results of subgroup analysis showed that both upregulated circRNAs (HR = 2.24, 95% Cl: 1.34–3.75, *I*^2^ = 0%) and downregulated circRNAs (HR = 0.61, 95% Cl: 0.45–0.83, *I*^2^ = 0%) were associated with poor BC prognosis. Collectively, the results of all relevant articles collected indicated that circRNAs showed good potential as possible clinical biomarkers of chemoresistance in BC patients.

## Introduction

Currently, the incidence of BC ranks second-highest among that of cancers worldwide, with 2,261,419 cases every year^1^. The incidence of BC is increasing year by year, and the age of onset is decreasing. Exploring new molecular markers of BC is beneficial for predicting prognosis accurately and monitoring curative effects. Therefore, finding an effective, rapid, noninvasive and specific marker is urgent and is crucial for the diagnosis, prognosis evaluation and drug resistance evaluation of BC^2,3^.

The choice of drugs for BC patients varies according to individual circumstances^4^. To date, the main treatments for BC are surgery, radiotherapy and chemotherapy^5^. Chemotherapy is a standard method for BC treatment^6^. There are many commonly used chemotherapy drugs for BC, including anthracyclines (doxorubicin, epirubicin, doxorubicin liposomes, etc.), paclitaxel drugs (paclitaxel, docetaxel, paclitaxel liposomes, and nab-paclitaxel) and fluorouracil (5-FU, capecitabine). In addition, there are targeted drugs such as trastuzumab and pertuzumab^7,8^ for BC. However, patients are developing resistance to conventional drugs. Chemotherapy resistance is one of the main reasons for clinical treatment failure and poor prognosis in BC patients^9^. This resistance might be due to alterations in several main regulatory pathways, such as *PI3K/AKT*^10–12^. Recently, some studies have found that certain circRNAs are strongly associated with resistance to a number of anticancer drugs, ranging from traditional chemotherapy drugs to targeted and immunotherapy drugs^13–17^.

CircRNAs are endogenous RNAs characterized by a covalent ring structure. Compared with other RNAs, circRNAs are less abundant, but circRNAs exhibit the advantage of high tissue specificity^18^. Recently, many researchers have indicated that certain circRNAs in different tumors might play essential roles in tumor cell proliferation, metastasis and drug resistance^19^. Several studies have suggested that circRNAs affect the development of drug resistance and prognosis of BC patients. Upregulated or downregulated circRNAs are involved in tumor growth and drug resistance, affecting the prognosis of breast cancer patients. Liang et al. showed that circKDM4C could inhibit BC proliferation and doxorubicin resistance in vitro, and this circRNA is a tumor suppressor in BC^20^. Wang et al. found that miR-142 regulated the *WWP1* and *PI3K/AKT* genes^10^. Circ-WAC could act as a sponge for miR-142 and decrease the inhibitory effect of miR-142 on its target *WWP1*. In addition, if triple-negative breast cancer patients expressed a high level of miR-142, their overall survival time was longer than that of other patients with low miR-142 expression. Additionally, Yang et al. showed that circ-CDR1as was involved in breast carcinogenesis and sensitivity to cisplatin in vivo. Knockdown of circ-CDR1as might increase the sensitivity of drug-resistant BC cells by reducing REGγ expression by eliminating the competition of miR-7^21^. Some articles have reported an association between changes in circRNAs and changes in drug resistance status in BC^11,20–32^. However, no article has summarized the specific mechanisms and modalities of circRNAs involved in BC drug resistance.

In the related meta-analysis, the involvement of circRNAs in BC was included, and we investigated the undiscovered prognostic value of circRNAs in BC. Several studies found that the expression of certain circRNAs was associated with increased drug resistance and a poor prognosis in BC patients^10,22,23,32^. Preclinical and clinical observational studies have shown that circRNA expression profiles can help identify patients at possible high risk for chemotherapy-resistant BC^11,33^. Therefore, we attempted to conduct a comprehensive systematic review and meta-analysis of published studies on circRNA-mediated chemoresistance in BC.

## Materials and methods

### Registration

We have registered the protocol on PROSPERO. Our registration number is CRD42022295180.

### Data search strategy

We searched all relevant articles through the PubMed, Embase, and Web of Science online databases that were published before 13 October 2022. The following entry words were used: (1) “Breast Neoplasms” or “Breast Cancer” or “BC." (2) “RNA, Circular” or “circRNAs” or “hsa circ”; (3) “Resistance, Drug” or “Drug resistance” or “chemoresistance.". The Preferred Reporting Items for Systematic Reviews and Meta-Analyses (PRISMA) guidelines were used to conduct search strategies.

### Inclusion and exclusion criteria

The selection criteria for inclusion in the literature were as follows: (1) studies that involved the effect of circRNAs on drug resistance or drug sensitivity in BC; (2) studies that collected clinical samples or involved in vitro preclinical analysis; and (3) studies that involved the effect of circRNA on the prognosis of BC. The criteria for exclusion were as follows: (1) duplicate studies; (2) reviews, editorials, opinions, case studies, and reports; unpublished materials, uninterpretable data, conference proceedings, or theses; (3) articles without complete information; (3) studies that did not indicate whether circRNA expression was upregulated or downregulated; (4) studies that did not include specific drug resistance changes; and (5) studies in languages other than English.

### Data extraction

Two researchers (Z.Z. and H.J.) extracted the data independently. When necessary, divergences were resolved by a third investigator (J.X.). The extracted information was as follows: (1) first author, publication year, circRNA, number of patients, detection methods for circRNAs, HR, CI; (2) follow-up time and outcomes; and (3) clinicopathological features, including TNM stage and T classification. When the results were not directly shown in the articles for HRs and 95% CIs, survival data were extracted from Kaplan‒Meier plots using Engauge Digitizer 4.1 software. The Excel program file of Tierney et al^34^. was then be used to calculate the HRs and 95% CIs.

### Quality assessment

Two independent investigators (Z.Z. and H.J.) used the Newcastle Ottawa Scale (NOS) to assess the quality of the articles for meta-analysis. If one study had a total score of > 6 points, it was considered high quality. When necessary, divergences were resolved by a third investigator (J.X.).

### Statistical analysis

Statistical analysis was performed using Stata 15.0. Data in the form of Kaplan‒Meier survival curves were converted to HRs and 95% CIs. Pooled outcome data were generated for forest plots to assess the prognostic value of circRNAs in BC. Heterogeneity tests were obtained by Cochran's Q test and Higgins *I*^2^. According to the rule, a random-effects model was used to generate pooled results if the *I*^2^ value was > 50%, and a fixed-effects model was used if the *I*^2^ value was <  = 50%. A p value < 0.05 was used to determine statistical significance.

## Results

### Selection of studies

**Figure 1:**
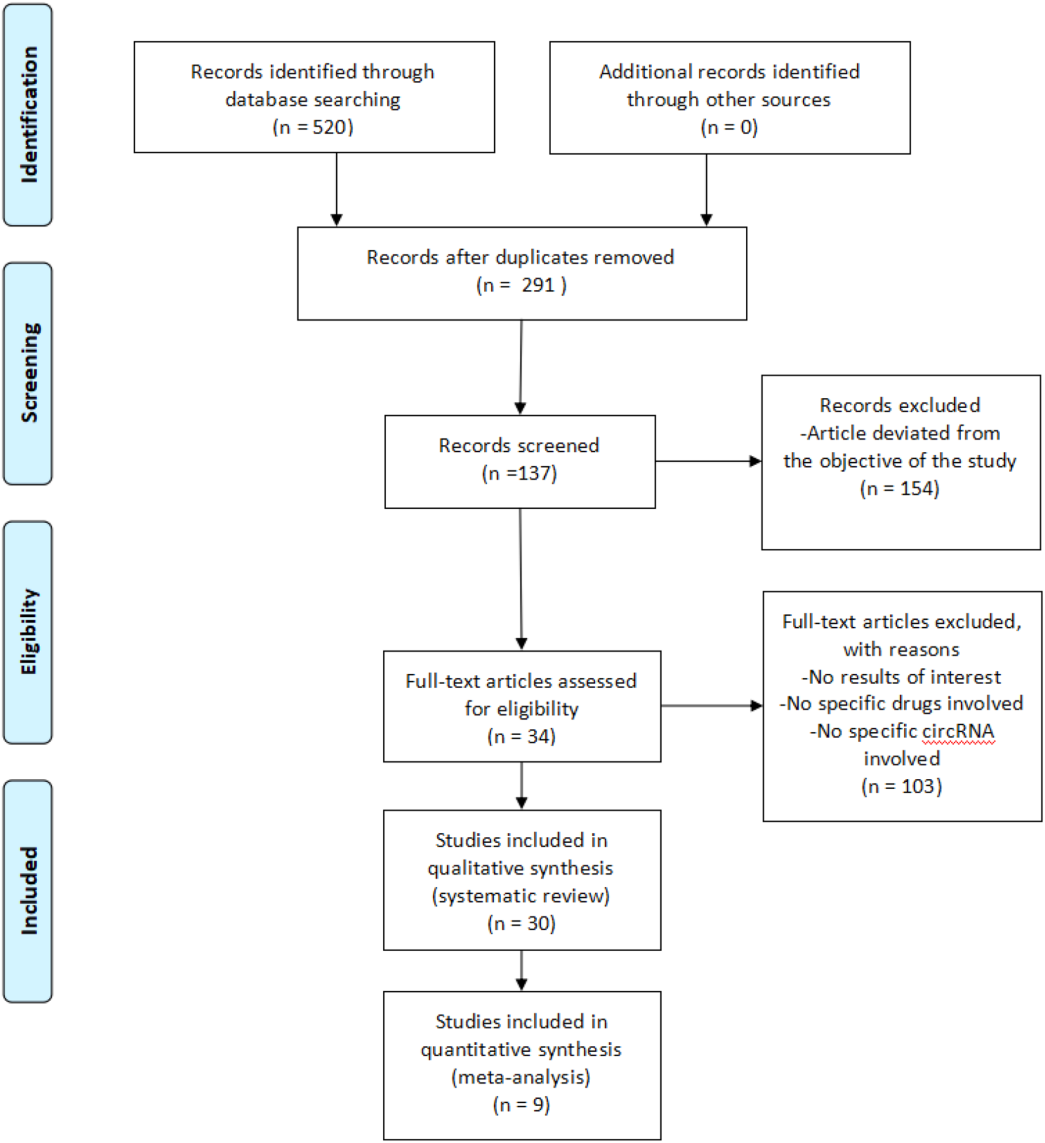
Flowchart of trial selection.

Figure 1 shows a flowchart for study selection. Through the search strategy, 520 articles were identified from PubMed, Embase, and Web of Science. After deduplication, screening criteria were used to review 291 potentially eligible studies. A careful selection of 137 articles was made by finalizing 34 full-text studies with available information according to PRISMA guidelines. Of these 34 articles, four studies were excluded since they were about other cancers. After multistep screening, the remaining 30 articles were used for systematic reviews^10–12,20–32,35–47^, of which nine were used for meta-analysis.

### Study characteristics and quality assessment

All included studies were collected until 13 October 2022 (the details of the description of the 30 included studies are shown in supplemental appendix 1). The 11 chemotherapy drugs used in the studies included 5-FU, lapatinib, adriamycin, doxorubicin, paclitaxel, cisplatin, monastrol, tamoxifen, docetaxel, trastuzumab, and oxaliplatin. Of these, paclitaxel is the most commonly used chemotherapeutic agent in clinical practice, while lapatinib and oxaliplatin are the least used. A total of 2077 BC tissue samples were included in the analysis. Seven of 30 studies documented clinical stage, including 187 in stage I, 464 in stage II, and 211 in stage III. Thirty studies used reverse transcription-polymerase chain reaction (RT‒qPCR) to detect circRNA, and only one study used the raw sequencing reads. Nine studies with survival curves were included in the meta-analysis, containing a total of 1962 individuals.

### Preclinical and clinical investigation of circRNA expression

A total of 17 different cell lines were used in 30 studies to explore circRNA expression and its association with drug resistance and associated pathways or proteins/axes. MCF-7 was the most commonly used cell line, while the U343 and U251 cell lines were the least commonly used. The experimental methods used in these studies include western blot, transfection and vector construction, flow cytometry, Transwell, ELISA, cytotoxicity assay, dual‑luciferase reporter assay, RNA pull‑down, RIP assay, IHC assay, RNase R treatment assay, 5-ethynyl-2′-deoxyuridine (EdU) assay, fluorescence in situ hybridization (FISH) assay, exosome tracing and blockade of exosome secretion.

After excluding duplicate circRNAs, our systematic review included a total of 30 different circRNAs, 28 of which were associated with increased drug resistance and a poor prognosis in breast cancer patients when their expression was upregulated, while only 2 were associated with increased drug resistance and a poor prognosis in breast cancer patients when their expression was downregulated.

### BC chemoresistance and drug‑regulated genetic pathways

In these 30 studies, a total of 32 circRNAs were reported, and excluding duplicate circRNAs, a total of 30 circRNAs were reported, and these circRNAs led to resistance to 11 drugs through 28 pathways or associated proteins/axes. (Table 1).

**Table 1:** Genetic pathways, proteins or axes involved in BC drug resistance.

Downregulated			Upregulated		
Drug	CircRNA	Pathway or associated protein/axis	Drug	CircRNA	Pathway or associated protein/axis
Doxorubicin	circ-LARP4	miR-761/p53, p21	5‐FU	circ-CDR1as	CCNE1
	circ-KDM4C	miR-548p/PBLD axis		circ-FBXL5	HMGA2
			Lapatinib	circ-MMP11	miR-153-3p/Anillin axis
			Adriamycin	circ-0006528	MAPK and PI3K/AKT
				circ-0001667	NCOA3
				circ-0006528	miR-1236-3p/CHD4 axis
				circ-0085495	miR-873-5p/integrinβ1 axis
				circ-0044556	miR‑145/NRAS axis
			Doxorubicin	circ-UBE2D2	miR-512-3p/CDCA3 axis
				circ-0092276	miR-348/ATG7 axis
			Paclitaxel	circ-RNF111	E2F3
				circ-ABCB10	Let-7a-5p/DUSP7 axis
				circ-WAC	WWP1, PI3K/AKT
				circ-HIPK3	HK2
				circ-AMOTL1	AKT
				circ-0006528	miR-1299/CDK8 axis
			Cisplatin	circ-CDR1as	miR-7/REGγ
				circUBAP2	miR-300/ASF1B axis chaperone/PI3K/AKT/mTOR axis
			Monastrol	circ-MTO1	TRAF4/Eg5 axis
			Tamoxifen	circ-0025202	miR-197-3p/HIPK3 axis
				circTRIM28	miR-409-3p/HMGA2 axis
				circMET	miR-204/AHR
				circ-0097922	miR-876-3p/ACTN4 axis
				circ-0025202	miR-182-5p/FOXO3a Axis
			Docetaxel	circ-EPHA3.1/circ-EPHA3.2/circ-ABCB1	PI3K-AKT/AGE-RAGE
			Trastuzumab	circCDYL2	HER2
				circ-0001598	miR-1184/PD-L1
			Oxaliplatin	circFAT1	miR-525-5p/SKA1 axis

### Findings of prognosis analysis

**Figure 2:**
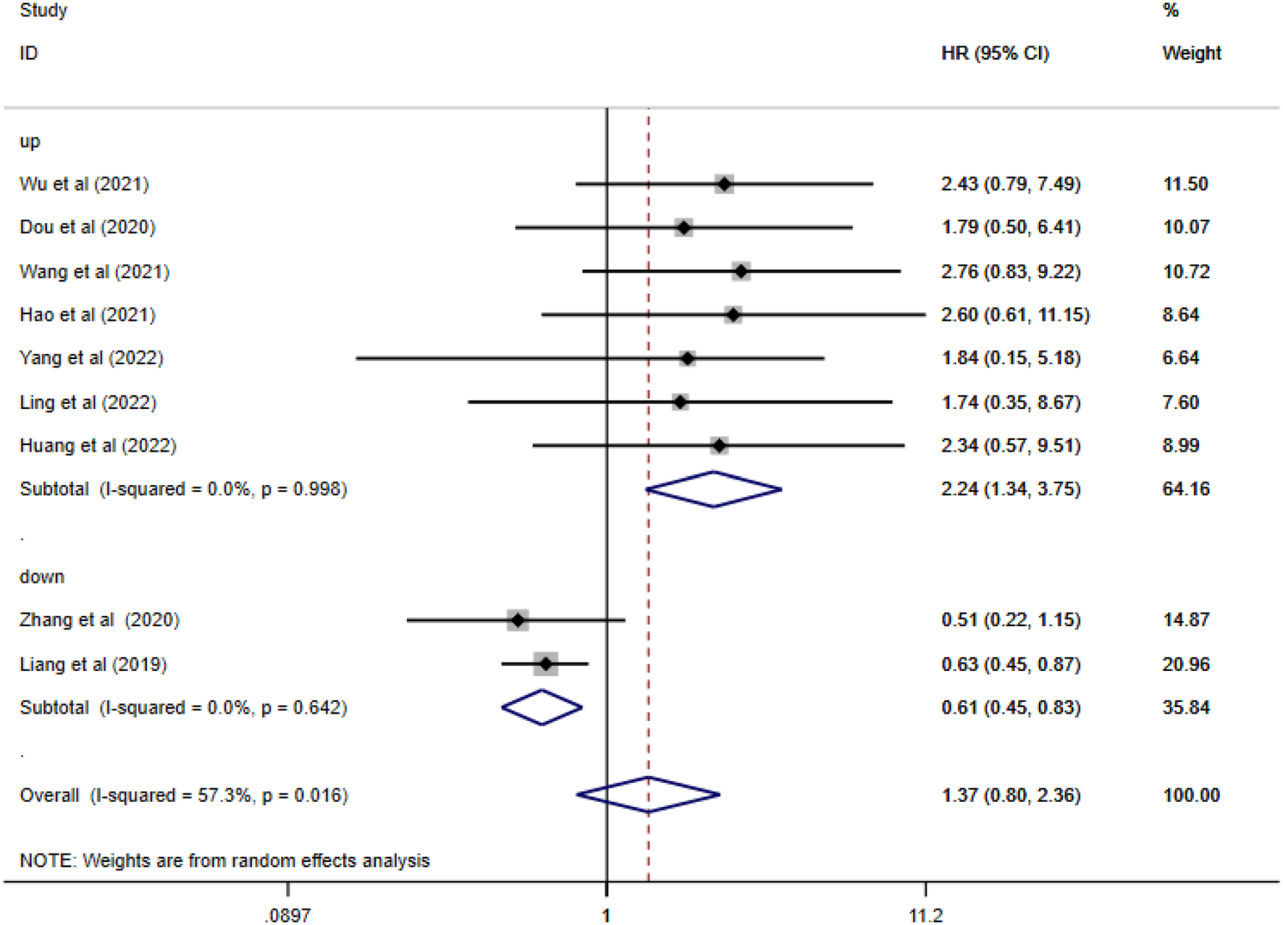
Pooled HRs for the overall survival of patients in the included studies.

Nine circRNAs were used for meta-analysis. Seven circRNAs were upregulated, and two were downregulated (Table 2 demonstrates details of prognostic research). The Newcastle–Ottawa Scale (NOS) was used to evaluate the quality of the included research (Table 3). The results showed that they all qualified for meta-analysis. The results of the meta-analysis showed that both the upregulated and downregulated groups were at risk for poor prognosis (HR = 1.37, 95% Cl: 0.80–2.36, *I*^2^ = 63.7%). There was significant heterogeneity between the studies. Therefore, we classified all circRNAs into "enhanced resistance"-related circRNAs and "attenuated resistance"-related circRNAs according to the expression of circRNAs. Subgroup analysis was performed according to the upregulation or downregulation of circRNAs. Interestingly, the heterogeneity was significantly reduced after performing a subgroup analysis (Fig. 2), which suggested that circRNAs could be used to determine the prognosis of BC patients (upregulated circRNAs (HR = 2.24, 95% Cl: 1.34–3.75, *I*^2^ = 0%) and downregulated circRNAs (HR = 0.61, 95% Cl: 0.45–0.83, *I*^2^ = 0%) were associated with poor BC prognosis.). All four circRNAs in the upregulated group were highly expressed in tumor tissues, and they affected gene pathways that promoted drug resistance in breast cancer cells, while the two circRNAs in the downregulated group were expressed at low levels in tumor tissues and affected gene pathways that inhibited proliferation, metastasis and drug resistance in breast cancer cells.

**Table 2:** Basic features of studies for prognostic analysis.

Author	Year	Country	CircRNA	Cancer type	High	Low	Methods	Regulation	follow up (month)	HR	CI
Wu et al.^22^	2021	China	circ-MMP11	BC	27	21	RT‒qPCR	Upregulated	60	2.43	0.79–7.49
Dou et al.^23^	2020	China	circ-UBE2D2	BC	33	33	RT‒qPCR	Upregulated	60	2.39	0.9–6.32
Wang et al.^26^	2021	China	circ-WAC	BC	45	45	RT‒qPCR	Upregulated	80	2.44	0.57–10.5
Hao et al.^10^	2021	China	circ-0006528	BC	32	31	RT‒qPCR	Upregulated	60	2.46	0.47–12.5
Zhang et al.^20^	2020	China	circ-LARP4	BC	142	141	RT‒qPCR	Downregulated	60	0.51	0.22–1.15
Liang et al.^32^	2019	China	circ-KDM4C	BC	474	587	RT‒qPCR	Downregulated	150	0.65	0.41–1.03
Yang et al.^40^	2022	China	circTRIM28	BC	32	32	RT‒qPCR	Upregulated	60	1.84	0.15–5.18
Ling et al.^43^	2022	China	circMET	BC	64	63	RT‒qPCR	Upregulated	120	1.74	0.35–8.67
Huang et al.^47^	2022	China	circ-0025202	BC	50	50	RT‒qPCR	Upregulated	80	2.34	0.57–9.51

**Table 3:** Quality assessment of included studies using the Newcastle Ottawa Scale checklist.

Author/Year	①	②	③	④	⑤	⑥	⑥	⑧	Total points
Wu et al. 2021^22^	1	1	1	1	1	1	1	0	7
Dou et al. 2021^23^	1	1	1	1	1	1	1	0	7
Zhang et al. 2019^26^	1	1	1	1	1	1	1	1	8
Wang et al. 2021^10^	1	1	1	1	1	1	1	0	7
Liang et al.2019^20^	1	1	1	1	1	1	1	1	8
Hao et al. 2021^32^	1	1	1	1	1	1	1	0	7
Yang et al.2022^40^	1	1	1	1	1	1	1	1	8
Ling et al.2022^43^	1	1	1	1	1	1	1	1	8
Huang et al.2022^47^	1	1	1	1	1	1	1	1	8

① Adequacy of case definition ② Number of cases ③ Representativeness of the cases ④ Ascertainment of relevant cancers ⑤ Ascertainment of detection method ⑥ CircRNA expression ⑦ Assessment of outcome ⑧ Adequate follow-up.

### Sensitivity analysis and publication bias

**Figure 3:**
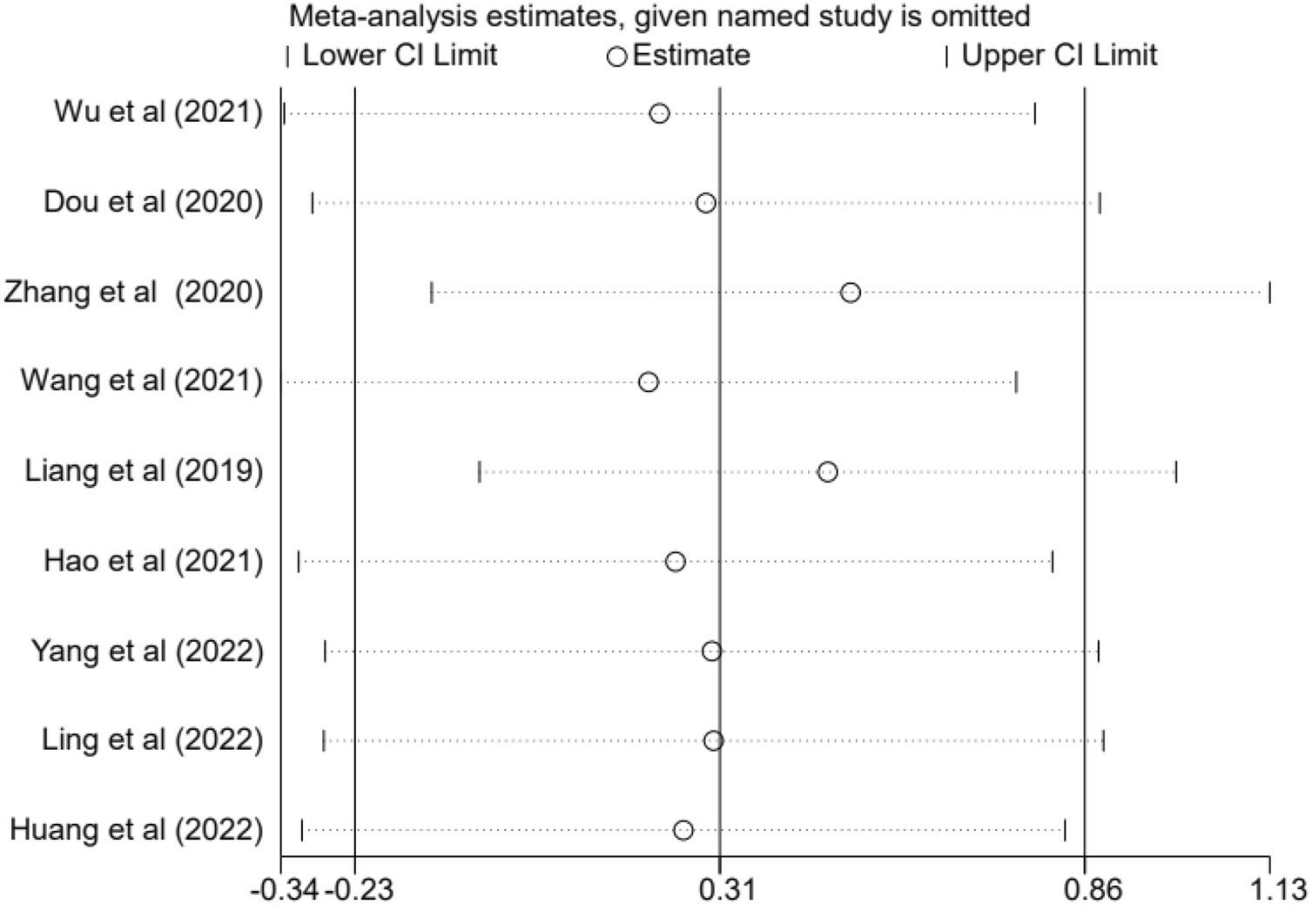
Sensitivity analysis for the involved studies.

**Figure 4:**
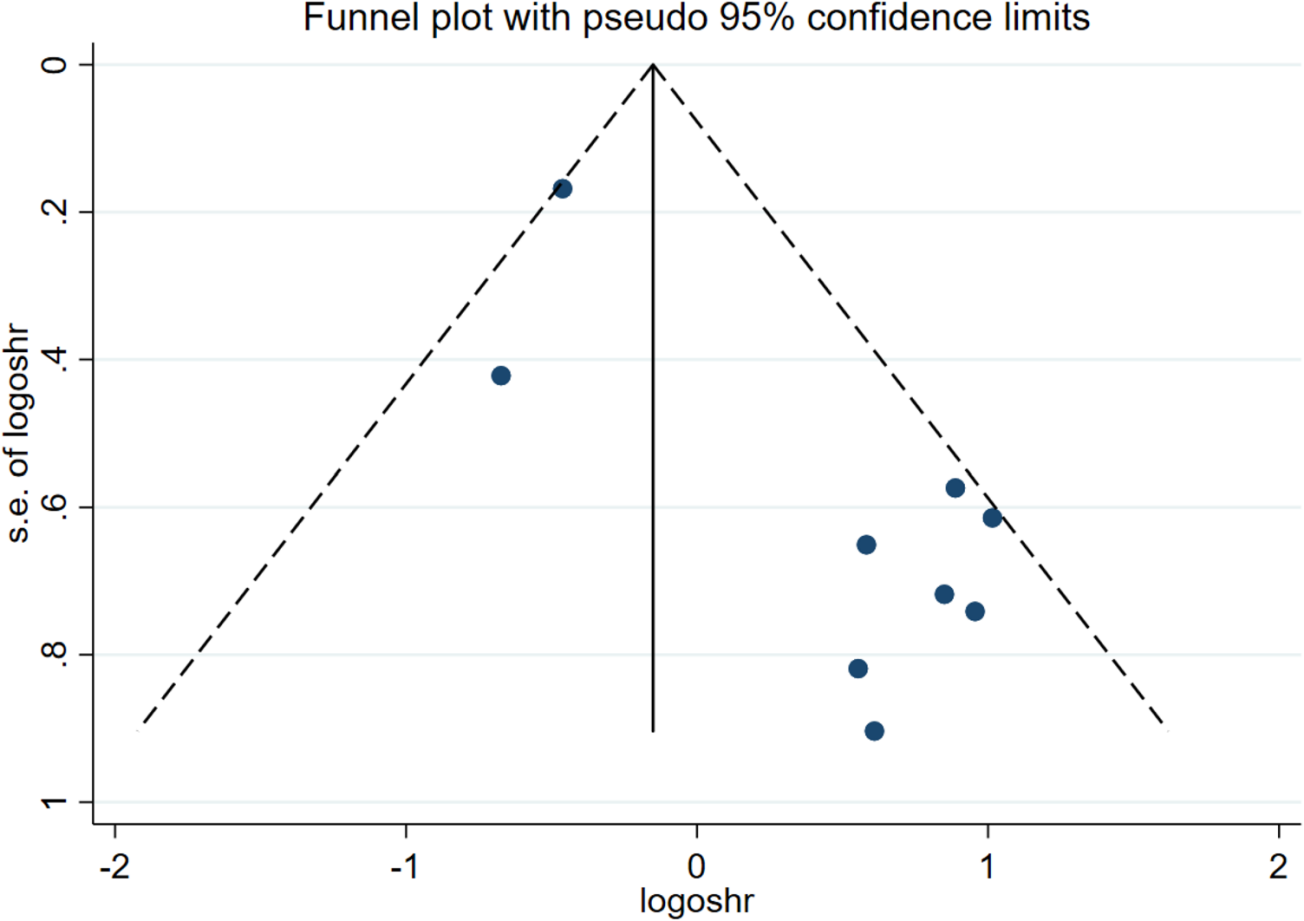
Funnel plot of publication bias related to the association between the expression of circRNAs and the prognosis of patients with BC.

**Figure 5:**
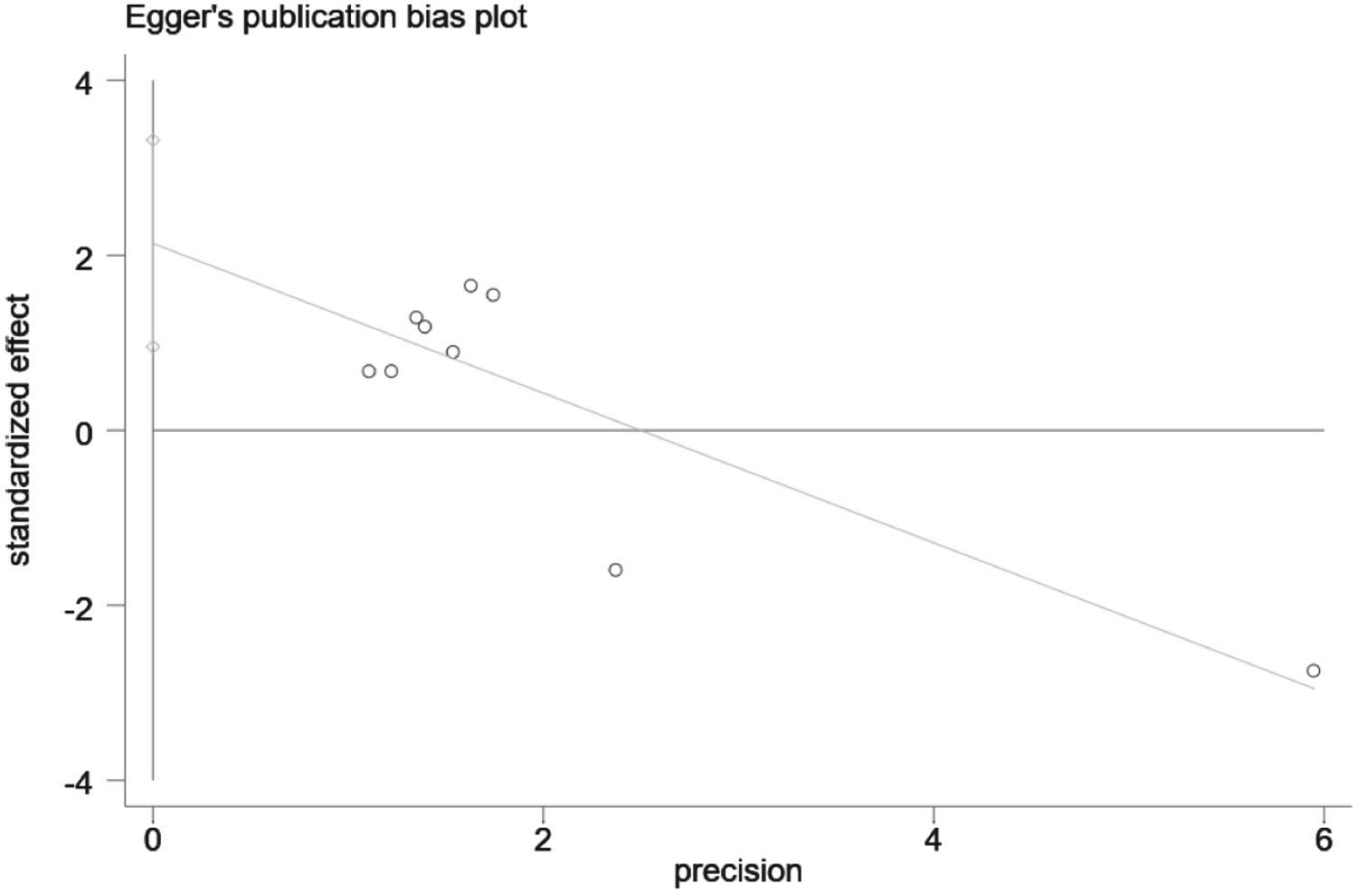
Egger's test for publication bias.

We also performed a sensitivity analysis for OS. No significant changes were observed compared to previous results after each study was removed (Fig. 3). In addition, we used funnel plots to assess publication bias. Each dot represents one study. Nine studies fell within the 95% confidence interval. The reason for the poor symmetry may be due to the inconsistent effect of circRNAs in the upregulated and downregulated groups (Fig. 4). Finally, we performed Begg's test, which showed *P* = 0.004 (< 0.05), and Egger's test suggested publication bias, which may be because far more circRNAs were upregulated than downregulated among the nine circRNAs (Fig. 5).

## Discussion

Studies have shown that abnormal circRNA expression is important in tumor cell proliferation, metastasis and cancer recurrence in BC patients^10–12,20–32,35–39^. Many studies have also confirmed that several specific circRNAs are consistently expressed in human tissues and blood. Therefore, circRNAs have the chance to be excellent biomarkers for BC diagnosis, prognosis and drug resistance assessment^3,33,48,49^.

Some studies have concentrated on the effects of circRNAs on chemoresistance in breast, cervical^50^, colorectal^51,52^, gastric^53,54^, lung^55^, oral^56^, ovarian^57^, pancreatic^58^ and prostate^59^ cancers. In this study, we collected relevant articles before 25 October 2021 and conducted a systematic review and meta-analysis, hoping to find clues about the value of circRNAs as biomarkers for BC prognosis. In the systematic review, studies incorporating 30 circRNAs, including 28 upregulated circRNAs and two downregulated circRNAs, were included. Most studies investigated only one circRNA, while only one study focused on more than one circRNA^12^. Our systematic review focused on pharmacological modulation pathways, including *MAPK, PI3K/AKT*, *AKT* and *AGE-RAGE,* in BC chemotherapy resistance and sensitivity.

Several studies have shown that target genes of upregulated circ-00006528 play a role in the *MAPK* and *PI3K/AKT* gene pathways. Further validation showed that the expression of circ-0006528 showed a negative correlation with miR-7-5p in adriamycin resistance. Another study showed a significant increase in both phosphorylated and total *AKT* protein in some circ-AMOTL1-overexpressing cells, suggesting that *AKT* might be a key factor in adjusting the resistance effect. Thus, circ-AMOTL1 affected the expression of proapoptotic (*BAX* and *BAK*) and antiapoptotic (BCL-2) factors associated with *AKT*^59^. This suggested that circ-AMOTL1 might be important in paclitaxel resistance in BC cells by affecting the *AKT* pathway, promoting antiapoptotic proteins and inhibiting proapoptotic proteins. In addition, data from a study showed that circ-ABCB1, circ-cEPHA3.1 and circ-EPHA3.2 might sponge several significantly expressed miRNAs related to drug resistance through the *PI3K-AKT* and AGE signaling pathways and lead to doxorubicin resistance^59^. They also found that the expression of RNA molecules transcribed from this region might be due to DNA amplification in doxorubicin-treated cells. These results are beneficial for subsequent research on the mechanisms of drug resistance in BC.

Nine circRNAs related to prognosis were included in the meta-analysis and were critical to the development of drug resistance. Among them, seven were upregulated (circ-MMP11, circ-WAC, circ-UBE2D2, circ-0006528, circTRIM28, circCDYL2, circ-0001598), and two were downregulated (circ-LARP4, circ-KDM4C). Certain cancer-related genes could increase susceptibility to breast cancer, leading to poorer survival rates. In our analysis, the results showed that the overall HR (95% CI) of upregulated circRNAs was 2.24 (1.34, 3.75), and that of downregulated circRNAs was 0.61 (0.45, 0.83), suggesting that both upregulated circRNAs and downregulated circRNAs could predict poorer cancer prognosis. It is worth noting that if circRNAs could be used as prognostic biomarkers of breast cancer, their clinical application prospects would be very broad. Other typical clinical indicators of tumor status are susceptible to change, but the expression of circRNAs is stable^60^. Steps should be taken to comprehensively assess the role of circRNAs as biomarkers for BC prognosis and drug-resistance assessment.

Some shortcomings must be acknowledged. First, in our study, all of the samples we collected were from Asian populations. Samples collected from a single source may not be able to distinguish between regional and racial differences and ethnic differences. Second, the method used to detect circRNAs was RT‒PCR, except for one study that used raw sequencing reads. The relative homogeneity of the methods used to detect circRNAs may affect the value of the assay results. Third, the meta-analysis involved a relatively small sample size and was limited by the number of available articles. In addition, the number of studies included in the meta-analysis was so low that the results of publication bias using funnel plots may not be meaningful.

Overall, circRNAs, as stably expressed molecules, are expected to be biomarkers for breast cancer prognosis. The relationship between circRNA expression and breast cancer features needs to be further investigated, and the practical value of circRNAs in evaluation BC drug resistance and prognosis needs to be further explored.

## Conclusion

Currently available evidence suggests that circRNAs might be considered potential prognostic biomarkers for BC patients and that there is a significant association between the expression of circRNAs and the prognosis of breast cancer patients. We anticipate that our findings might contribute to BC treatment.

## Supplementary Information

Supplementary Information 1.

## Data Availability

Data are available in a public, open access repository. The corresponding author of this paper can provide relevant information supporting the conclusions of this study if needed.

## Abbreviations

CircRNAs: Circular RNAs
BC: Breast cancer
CI: Confidence interval
HR: Hazard ratio
PRISMA: Preferred items for systematic reviews and meta-analyses
RT-PCR: Real-time reverse transcription-polymerase chain reaction
MiRNA: MicroRNA
OS: Overall survival

## References

[1] Organization, W. H. *Breast Cancer: Estimated Incidence, Mortality and Prevalence Worldwide in 2021.* (2021).

[2] Li Y (2019). Extracellular vesicles long RNA sequencing reveals abundant mRNA, circRNA, and lncRNA in human blood as potential biomarkers for cancer diagnosis. Clin. Chem..

[3] Tian T (2021). Circular RNA: A potential diagnostic, prognostic, and therapeutic biomarker for human triple-negative breast cancer. Mol. Therap. Nucleic acids.

[4] Trayes KP, Cokenakes SEH (2021). Breast cancer treatment. Am. Fam. Phys..

[5] Fisusi FA, Akala EO (2019). Drug combinations in breast cancer therapy. Pharm. Nanotechnol..

[6] Montemurro F, Nuzzolese I, Ponzone R (2020). Neoadjuvant or adjuvant chemotherapy in early breast cancer?. Expert Opin. Pharmacother..

[7] Jabbarzadeh Kaboli P (2020). Akt-targeted therapy as a promising strategy to overcome drug resistance in breast cancer—A comprehensive review from chemotherapy to immunotherapy. Pharmacol. Res..

[8] Liyanage PY (2019). Nanoparticle-mediated targeted drug delivery for breast cancer treatment. Biochim. Biophys. Acta. Rev. Cancer.

[9] Afzal M (2021). Nanomedicine in treatment of breast cancer—A challenge to conventional therapy. Semin. Cancer Biol..

[10] Wang L (2021). CircWAC induces chemotherapeutic resistance in triple-negative breast cancer by targeting miR-142, upregulating WWP1 and activating the PI3K/AKT pathway. Mol. Cancer.

[11] Gao D (2017). Screening circular RNA related to chemotherapeutic resistance in breast cancer. Epigenomics.

[12] Huang P (2021). A comprehensive RNA study to identify circRNA and miRNA biomarkers for docetaxel resistance in breast cancer. Front. Oncol..

[13] Sang Y (2019). circRNA_0025202 regulates tamoxifen sensitivity and tumor progression via regulating the miR-182-5p/FOXO3a axis in breast cancer. Mol. Therap. J. Am. Soc. Gene Therap..

[14] Huang XY (2020). Circular RNA circMET drives immunosuppression and anti-PD1 therapy resistance in hepatocellular carcinoma via the miR-30-5p/snail/DPP4 axis. Mol. Cancer.

[15] Chen DL (2021). The circular RNA circDLG1 promotes gastric cancer progression and anti-PD-1 resistance through the regulation of CXCL12 by sponging miR-141-3p. Mol. Cancer.

[16] Wei W (2021). Circ0008399 interaction with WTAP promotes assembly and activity of the m(6)A methyltransferase complex and promotes cisplatin resistance in bladder cancer. Cancer Res..

[17] Huang KB (2021). Circular RNA circSNX6 promotes sunitinib resistance in renal cell carcinoma through the miR-1184/GPCPD1/ lysophosphatidic acid axis. Cancer Lett..

[18] Lei M, Zheng G, Ning Q, Zheng J, Dong D (2020). Translation and functional roles of circular RNAs in human cancer. Mol. Cancer.

[19] Wang J, Zhang Y, Liu L, Yang T, Song J (2021). Circular RNAs: New biomarkers of chemoresistance in cancer. Cancer Biol. Med..

[20] Liang Y (2019). circKDM4C suppresses tumor progression and attenuates doxorubicin resistance by regulating miR-548p/PBLD axis in breast cancer. Oncogene.

[21] Yang W (2019). Silencing CDR1as enhances the sensitivity of breast cancer cells to drug resistance by acting as a miR-7 sponge to down-regulate REG gamma. J. Cell Mol. Med..

[22] Wu X, Ren Y, Yao R, Zhou L, Fan R (2021). Circular RNA circ-MMP11 contributes to lapatinib resistance of breast cancer cells by regulating the miR-153–3p/ANLN Axis. Front. Oncol..

[23] Dou D (2020). CircUBE2D2 (hsa_circ_0005728) promotes cell proliferation, metastasis and chemoresistance in triple-negative breast cancer by regulating miR-512–3p/CDCA3 axis. Cancer Cell Int..

[24] Cui Y, Fan J, Shi W, Zhou Z (2021). Circ_0001667 knockdown blocks cancer progression and attenuates adriamycin resistance by depleting NCOA3 via releasing miR-4458 in breast cancer. Drug Dev. Res..

[25] Zhu M, Wang Y, Wang F, Li L, Qiu X (2021). CircFBXL5 promotes the 5-FU resistance of breast cancer via modulating miR-216b/HMGA2 axis. Cancer cell Int..

[26] Zhang X, Su X, Guo Z, Jiang X, Li X (2020). Circular RNA La-related RNA-binding protein 4 correlates with reduced tumor stage, as well as better prognosis, and promotes chemosensitivity to doxorubicin in breast cancer. J. Clin. Lab. Anal..

[27] Zang H, Li Y, Zhang X, Huang G (2020). Circ-RNF111 contributes to paclitaxel resistance in breast cancer by elevating E2F3 expression via miR-140-5p. Thorac. cancer.

[28] Yang W (2020). Circ-ABCB10 contributes to paclitaxel resistance in breast cancer through Let-7a-5p/DUSP7 axis. Cancer Manag Res..

[29] Ni J, Xi X, Xiao S, Xiao X (2021). Silencing of circHIPK3 sensitizes paclitaxel-resistant breast cancer cells to chemotherapy by regulating HK2 through targeting miR-1286. Cancer Manag. Res..

[30] Liu G (2020). Circ_0006528 contributes to paclitaxel resistance of breast cancer cells by regulating miR-1299/CDK8 axis. Onco. Targets. Ther..

[31] Li H, Li Q, He S (2021). Hsa_circ_0025202 suppresses cell tumorigenesis and tamoxifen resistance via miR-197–3p/HIPK3 axis in breast cancer. World J. Surg. Oncol..

[32] Hao J, Du X, Lv F, Shi Q (2021). Knockdown of circ_0006528 suppresses cell proliferation, migration, invasion, and adriamycin chemoresistance via regulating the miR-1236-3p/CHD4 axis in breast cancer. J. Surg. Res..

[33] Huang R, Yu H, Zhong X (2021). Identification of novel CircRNA-miRNA-mRNA regulatory network and its prognostic prediction in breast cancer. Evid. Complement. Altern. Med. eCAM.

[34] Tierney JF, Stewart LA, Ghersi D, Burdett S, Sydes MR (2007). Practical methods for incorporating summary time-to-event data into meta-analysis. Trials.

[35] Ma J (2019). Posttranscriptional regulation of AKT by circular RNA angiomotin-like 1 mediates chemoresistance against paclitaxel in breast cancer cells. Aging Us.

[36] Liu Y, Dong Y, Zhao L, Su L, Luo J (2018). Circular RNA-MTO1 suppresses breast cancer cell viability and reverses monastrol resistance through regulating the TRAF4/Eg5 axis. Int. J. Oncol..

[37] Xie H, Zheng R (2021). Circ_0085495 knockdown reduces adriamycin resistance in breast cancer through miR-873-5p/integrin β1 axis. Anticancer Drugs.

[38] Xiong D (2020). The latest overview of circRNA in the progression, diagnosis, prognosis, treatment, and drug resistance of hepatocellular carcinoma. Front. Oncol..

[39] Wang Q (2021). Hsa_circ_0092276 promotes doxorubicin resistance in breast cancer cells by regulating autophagy via miR-348/ATG7 axis. Transl. Oncol..

[40] Yang S (2022). Knockdown circTRIM28 enhances tamoxifen sensitivity via the miR-409-3p/HMGA2 axis in breast cancer. Reprod Boil. Endocrinol. RB E.

[41] Wang L, Yang X, Zhou F, Sun X, Li S (2022). Circular RNA UBAP2 facilitates the cisplatin resistance of triple-negative breast cancer via microRNA-300/anti-silencing function 1B histone chaperone/PI3K/AKT/mTOR axis. Bioengineered.

[42] Liu J (2022). Circular RNA circMET contributes to tamoxifen resistance of breast cancer cells by targeting miR-204/AHR signaling. Biochem. Biophys. Res. Commun..

[43] Ling Y (2022). circCDYL2 promotes trastuzumab resistance via sustaining HER2 downstream signaling in breast cancer. Mol. Cancer.

[44] Liang X, Liu X, Song Z, Zhu J, Zhang J (2022). Hsa_circ_0097922 promotes tamoxifen resistance and cell malignant behaviour of breast cancer cells by regulating ACTN4 expression via miR-876-3p. Clin. Exp. Pharmacol. Physiol..

[45] Chen J (2022). CircRNA_0044556 diminishes the sensitivity of triple-negative breast cancer cells to adriamycin by sponging miR-145 and regulating NRAS. Mol. Med. Rep..

[46] Sang Y (2021). circRNA_0025202 regulates tamoxifen sensitivity and tumor progression via regulating the miR-182-5p/FOXO3a axis in breast cancer. Mol. Therap J Am. Soc. Gene Therap..

[47] Huang L, Ma J, Cui M (2021). Circular RNA hsa_circ_0001598 promotes programmed death-ligand-1-mediated immune escape and trastuzumab resistance via sponging miR-1184 in breast cancer cells. Immunol. Res..

[48] Misir S, Hepokur C, Aliyazicioglu Y, Enguita FJ (2021). Biomarker potentials of miRNA-associated circRNAs in breast cancer (MCF-7) cells: An in vitro and in silico study. Mol. Biol. Rep..

[49] Zhang HD, Jiang LH, Sun DW, Hou JC, Ji ZL (2018). CircRNA: A novel type of biomarker for cancer. Breast cancer Tokyo Japan.

[50] Chen M (2019). circMTO1 promotes tumorigenesis and chemoresistance of cervical cancer via regulating miR-6893. Biomed. Pharmacother..

[51] Jian X (2020). Hsa_circ_001680 affects the proliferation and migration of CRC and mediates its chemoresistance by regulating BMI1 through miR-340. Mol. Cancer.

[52] Wang X (2020). Exosome-delivered circRNA promotes glycolysis to induce chemoresistance through the miR-122-PKM2 axis in colorectal cancer. Mol. Oncol..

[53] Liu S, Wu M, Peng M (2020). Circ_0000260 regulates the development and deterioration of gastric adenocarcinoma with cisplatin resistance by upregulating MMP11 via targeting MiR-129-5p. Cancer Manag. Res..

[54] Liu Y, Xu J, Jiang M, Ni L, Ling Y (2020). CircRNA DONSON contributes to cisplatin resistance in gastric cancer cells by regulating miR-802/BMI1 axis. Cancer Cell Int..

[55] Zheng S, Wang C, Yan H, Du Y (2021). Blocking hsa_circ_0074027 suppressed non-small cell lung cancer chemoresistance via the miR-379-5p/IGF1 axis. Bio-engineered.

[56] Qiu F (2021). Blocking circ-SCMH1 (hsa_circ_0011946) suppresses acquired DDP resistance of oral squamous cell carcinoma (OSCC) cells both in vitro and in vivo by sponging miR-338-3p and regulating LIN28B. Cancer Cell Int..

[57] Zheng Y, Li Z, Yang S, Wang Y, Luan Z (2020). CircEXOC6B suppresses the proliferation and motility and sensitizes ovarian cancer cells to paclitaxel through miR-376c-3p/FOXO3 Axis. Cancer Biother. Radiopharm..

[58] Xie W (2020). Emerging roles of long noncoding RNAs in chemoresistance of pancreatic cancer. Semin. Cancer Biol..

[59] Shen Z, Zhou L, Zhang C, Xu J (2020). Reduction of circular RNA Foxo3 promotes prostate cancer progression and chemoresistance to docetaxel. Cancer Lett..

[60] Zhao H (2021). The circRNA_102911/miR-129-5p/SOX6 axis is involved with T lymphocyte immune function in elderly patients with laparoscopic left hepatectomy for hepatolithiasis. Exp. Ther. Med..

