# Supplementary material for: Current evidence on circRNAs as potential theranostic markers for detecting chemoresistance in breast cancer: a systematic review and meta‑analysis
Source: Sci Rep. 2022 Dec 20;12:22016. doi: 10.1038/s41598-022-26220-z (PMC9768200; doi:10.1038/s41598-022-26220-z)
Supplement: Supplementary file 1 — Supplementary Information 1. [file 41598_2022_26220_MOESM1_ESM.pdf]

Current Evidence on CircRNAs as Potential Theranostic Markers for Detecting Chemoresistance in Breast Cancer: A Systematic Review and Meta-Analysis

Zixin Zhu[1†], Hui Jiang[1†], Jingling Xie[1], Xinrui Jin[1], Baolin Li[1], Jinbo Liu[1#]

[1] Department of Laboratory Medicine, the Affiliated Hospital of Southwest Medical University, 25 Taiping Street, Luzhou, 646000, Sichuan, P.R. China.

Zixin Zhu and Hui Jiang shared their first authorship and contributed equally to this work.

Tel: +86 0830 3165730

Fax: +86 0830 3165730

*Correspondence: Jinbo Liu: liujb7203@swmu.edu.cn

Appendix 1. Description of the 30 included studies

Study	Country	Chemotherapy	Samples (drug-resistant/ drug-sensitive)	Clinical stage	CircRNA	Cell line	CircRNAs profiling platform	Pathway or Associated protein/axis
Wei et al.[1] (2019)	China/Asian	5-FU	NR	NR	circ-CDR1as	MCF-7, MDA-MB-231	RT-PCR-SYBR (Applied Biosystems, USA)	CCNE1
Wu et al.[2] （2021）	China/Asian	Lapatinib	27/21	NR	circ-MMP11	MDA-MB-231, MCF-7	RT-PCR-SYBR （Applied Biosystems, Foster City, CA, USA)	miR-153-3p/Anillin axis
Gao et al.[3] (2017)	China/Asian	Adriamycin	20/20	NR	circ-0006528	MCF-7, MDA-MB-231	RT-PCR-SYBR （Applied Biosystems， USA）	MAPK and PI3K/AKT
Dou et al.[4] (2020)	China/Asian	Doxorubicin	33/33	TNM1 = TNM2 = TNM3 = 30	circ-UBE2D2	BT-549, SUM-159, MDA-MB-231, MDA-MB-468, HCC38	RT-PCR-SYBR (Applied Biosystems, Foster City, CA, USA)	miR-512-3p/CDCA 3 axis
Cui et al.[5] (2021)	China/Asian	Adriamycin	27/34	TNM1: TNM2:	circ-0001667	MCF-7, MDA-MB-231,	RT-PCR-SYBR (Applied	NCOA3

				TNM3 = 18:33:10		MCF-7, MDA-MB-231	Biosystems; Foster City, CA, USA)	
Zhu et al.[6] (2021)	China/Asian	5-FU	NR	TNM1+2：TNM3 = 26:13	circ-FBXL5	MDA-MB-453, MDA-MB-231	RT-PCR-SYBR (Applied Biosystems 7500 system)	HMGA2
Zhang et al.[7] (2020)	China/Asian	Doxorubicin	283	TNM1: TNM2: TNM3 = 67:155:61	circ-LARP4	MCF-7, MDA-MB-231	RT-PCR-SYBR（Bio-Rad）	miR-761/p53, p21
Zang et al.[8] (2020)	China/Asian	Paclitaxel	30/30	NR	circ-RNF111	MCF-7, MDA-MB-231	RT-PCR-SYBR (Applied Biosystems, Foster City, CA, USA)	E2F3
Yang et al.[9] (2019)	China/Asian	Cisplatin	90	NR	circ-CDR1as	MCF-7, SKBR-3, MDA-MB-231, MDA-MB-468, HCC-1937	RT-PCR (ABI, Austin, TX, USA)	miR-7/REGγ
Yang et al.[10] (2020)	China/Asian	Paclitaxel	30/30	NR	circ-ABCB10	MDA-MB-468, MDA-MB-453, MCF-7, MDA-MB-231	RT-PCR-SYBR	Let-7a-5p/DUSP7 axis

Wang et al.[11] (2021)	China/Asian	Paclitaxel	45/45	NR	circ-WAC	MDA-MB-231, MDA-MB-468, HCC-1937, MDA-MB-361, MCF-7, MCF-10A	RT-PCR	WWP1, PI3K/AKT
Ni et al.[12] (2021)	China/Asian	Paclitaxel	76	NR	circ-HIPK3	MDA-MB-231, MCF-7	RT-PCR- SYBR（Bio-Rad）	HK2
Ma et al.[13] (2019)	China/Asian	Paclitaxel	NR	NR	circ-AMOTL 1	MDA-MB-231	RT-PCR-SYBR（Bio-Rad）	AKT
Liu et al.[14] (2018)	China/Asian	Monastrol	NR	NR	circ-MTO1	MDA-MB-231, MCF-7, MDA-MB-453, SKBR-3, T47D, MDA-MB-468	RT-PCR (Applied Biosystems; Thermo Fisher Scientific, Inc.)	TRAF4/Eg5 axis
Liu et al.[15] (2020)	China/Asian	Paclitaxel	15/33	NR	circ-0006528	MCF10A, BT-549， ZR-75-30	RT-PCR-SYBR（Applied Biosystems, Foster City, CA, USA)	miR-1299/CDK8 axis
Liang et al.[16] (2019)	China/Asian	Doxorubicin	587/474	NR	circ-KDM4C	MDA-MB-231, MCF-7	RT-PCR（SYBR green PCR mix，Takara）	miR-548p/PBLD axis
Li et al.[17] (2021)	China/Asian	Tamoxifen	32	NR	circ-0025202	T47D, MCF7, MCF-10A	RT-PCR（SYBR green PCR mix，Takara）	miR-197-3p/HIPK3 axis

Hao et al.[18] (2021)	China/Asian	Adriamycin	32/31	NR	circ-0006528	MCF-7, MDA-MB-231, MCF-10A	RT-PCR（SYBR green PCR Master Mix，Thermo Fisher Scientific)	miR-1236-3p/CHD4 axis
Xie et al.[19] (2020)	China/Asian	Adriamycin	NR	NR	circ-0085495	MCF-7, MDA-MB-231	RT-PCR（SYBR green PCR Master Mix，Thermo Fisher Scientific)	miR-873-5p/integrin β1 axis
Wang et al.[20] (2021)	China/Asian	Doxorubicin	NR	NR	circ-0092276	MCF-7, MDA-MB-468,	RT-qPCR(SYBR green PCR mix，Takara)	miR-348/ATG7 axis
Huang et al.[21] (2021)	China/Asian	Docetaxel	NR	NR	circ-EPHA3.1/circ-EPHA3.2/circ-ABCB1	MDA-MB-231, MCF-7	The raw sequencing reads (Tophat2 software)	PI3K-AKT/AGE-RAGE
Yang et al.[22] (2022)	China/Asian	Tamoxifen	32/32	TNM1+2：TNM3 = 29:35	circTRIM28	MCF7, MDA-MB-231, MCF10A	RT-qPCR(SYBR green PCR mix，Takara)	miR-409-3p/HMGA2 axis
Wang et al.[23] (2022)	China/Asian	Cisplatin	57	NR	circUBAP2	HCC1937, BT-549, MDA-MB-436	RT-qPCR (Applied Biosystems; Thermo Fisher Scientific, Inc.)	miR-300/ASF1B axis, 1B histone chaperone/PI3K/AKT/mTOR axis
Liu et al.[24] (2022)	China/Asian	Tamoxifen	NR	NR	circMET	MCF7, T47D	RT-qPCR (SYBR Premix Ex TaqII , Takara, Japan)	miR-204/AHR

Ling et al[25] (2022)	China/Asian	Trastuzumab	64/63	NR	circCDYL2	BT474-R, SK-BR-3-R	RT-qPCR	HER2
Liang et al.[26] (2022)	China/Asian	Tamoxifen	46	NR	circ-0097922	MCF7, T47D, MCT-10A, HEK293T, CL-0005	RT-qPCR（SYBR green PCR mix，Takara）	miR-876-3p/ACTN4 axis
Chen et al.[27] (2022)	China/Asian	Adriamycin	40	NR	circ-0044556	MCF-10A, MDA-MB-231, MDA-MB-453, MDA-MB-157, BT549	RT-qPCR(Applied Biosystems; Thermo Fisher Scientific, Inc.)	miR-145/NRAS axis
Yao et al.[28] (2022)	China/Asian	Oxaliplatin	66	TNM1: TNM2: TNM3 = 6:55:5	circFAT1	MCF-10A, BT474, MCF-7, T47D	RT-qPCR(Applied Biosystems)	miR-525-5p/SKA1 axis
Sang et al.[29] (2022)	China/Asian	Tamoxifen	330	TNM1: TNM2: TNM3 = 11:162:57	circ-0025202	MCF7, T47D, the HEK293T	RT-qPCR(SYBR Premix Ex TaqII , Takara)	miR-182-5p/FOXO3a Axis
Huang et al.[30] (2022)	China/Asian	Trastuzumab	50/50	NR	circ-0001598	SKBR-3, BT474	RT-qPCR(Quant-Studio 3 machine, Applied Biosystems, Foster City, CA, USA)	miR-1184/PD-L1

Appendix 2. Search strategy

#1

Search: ("Breast Neoplasms"[Mesh]) OR (((((((((((((((((((((((((((((((((((((Breast Neoplasm) OR (Neoplasm, Breast)) OR (Breast Tumors)) OR (Breast Tumor)) OR (Tumor, Breast)) OR (Tumors, Breast)) OR (Neoplasms, Breast)) OR (Breast Cancer)) OR (Cancer, Breast)) OR (Mammary Cancer)) OR (Cancer, Mammary)) OR (Cancers, Mammary)) OR (Mammary Cancers)) OR (Malignant Neoplasm of Breast)) OR (Breast Malignant Neoplasm)) OR (Breast Malignant Neoplasms)) OR (Malignant Tumor of Breast)) OR (Breast Malignant Tumor)) OR (Breast Malignant Tumors)) OR (Cancer of Breast)) OR (Cancer of the Breast)) OR (Mammary Carcinoma, Human)) OR (Carcinoma, Human Mammary)) OR (Carcinomas, Human Mammary)) OR (Human Mammary Carcinomas)) OR (Mammary Carcinomas, Human)) OR (Human Mammary Carcinoma)) OR (Mammary Neoplasms, Human)) OR (Human Mammary Neoplasm)) OR (Human Mammary Neoplasms)) OR (Neoplasm, Human Mammary)) OR (Neoplasms, Human Mammary)) OR (Mammary Neoplasm, Human)) OR (Breast Carcinoma)) OR (Breast Carcinomas)) OR (Carcinoma, Breast)) OR (Carcinomas, Breast))

#2

Search: ("RNA, Circular"[Mesh]) OR ((((((((((((circRNAs) OR (Closed Circular RNA)) OR (Circular RNA, Closed)) OR (RNA, Closed Circular)) OR (Circular RNA)) OR (Circular RNAs)) OR (RNAs, Circular)) OR (circRNA)) OR (Circular Intronic RNA)) OR (Intronic RNA, Circular)) OR (RNA, Circular Intronic)) OR (ciRNA))

#3

Search: ("Drug Resistance"[Mesh]) OR (((((((((((((Resistance, Drug) OR (chemosensitivity)) OR (chemosensitize)) OR (chemotherapeutic)) OR (sensitivity)) OR (chemoresistance)) OR (resistance)) OR (sensitize)) OR (chemotherapeutic sensitivity)) OR (therapeutic outcome)) OR (chemotherapy resistance)) OR (chemotherapy resistant)) OR (chemotherapy))

1. #1 AND #2 AND #3
2. #1 AND #2
3. #1 OR #2 OR #3
4. #2 AND #3

Reference

1.Yang, W. *et al.* Inhibition of circular RNA CDR1as increases chemosensitivity of 5-FU-resistant BC cells through up-regulating miR-7. *Journal of cellular and molecular medicine* **23,** 3166-3177, doi:10.1111/jcmm.14171 (2019).

2.Wu, X., Ren, Y., Yao, R., Zhou, L. & Fan, R. Circular RNA circ-MMP11 Contributes to Lapatinib Resistance of Breast Cancer Cells by Regulating the miR-153-3p/ANLN Axis. *Frontiers in oncology* **11**, doi:10.3389/fonc.2021.639961 (2021).

3.Gao, D. *et al.* Screening circular RNA related to chemotherapeutic resistance in breast cancer. *Epigenomics* **9,** 1175-1188, doi:10.2217/epi-2017-0055 (2017).

4.Dou, D. *et al.* CircUBE2D2 (hsa_circ_0005728) promotes cell proliferation, metastasis and chemoresistance in triple-negative breast cancer by regulating miR-512-3p/CDCA3 axis. *Cancer Cell International* **20**, doi:10.1186/s12935-020-01547-7 (2020).

5.Cui, Y., Fan, J., Shi, W. & Zhou, Z. Circ_0001667 knockdown blocks cancer progression and attenuates adriamycin resistance by depleting NCOA3 via releasing miR-4458 in breast cancer. *Drug Development Research*, doi:10.1002/ddr.21845 (2021).

6.Zhu, M., Wang, Y., Wang, F., Li, L. & Qiu, X. CircFBXL5 promotes the 5-FU resistance of breast cancer via modulating miR-216b/HMGA2 axis. *Cancer cell international* **21**, doi:10.1186/s12935-021-02088-3 (2021).

7.Zhang, X., Su, X., Guo, Z., Jiang, X. & Li, X. Circular RNA La-related RNA-binding protein 4 correlates with reduced tumor stage, as well as better prognosis, and promotes chemosensitivity to doxorubicin in breast cancer. *Journal of clinical laboratory analysis* **34**, doi:10.1002/jcla.23272 (2020).

8.Zang, H., Li, Y., Zhang, X. & Huang, G. Circ-RNF111 contributes to paclitaxel resistance in breast cancer by elevating E2F3 expression via miR-140-5p. *Thoracic cancer* **11,** 1891-1903, doi:10.1111/1759-7714.13475 (2020).

9.Yang, W. *et al.* Silencing CDR1as enhances the sensitivity of breast cancer cells to drug resistance by acting as a miR-7 sponge to down-regulate REG gamma. *Journal of cellular and molecular medicine* **23,** 4921-4932, doi:10.1111/jcmm.14305 (2019).

10.Yang, W. *et al.* Circ-ABCB10 Contributes to Paclitaxel Resistance in Breast Cancer Through Let-7a-5p/DUSP7 Axis. *Cancer management and research* **12,** 2327-2337, doi:10.2147/cmar.S238513 (2020).

11.Wang, L. *et al.* CircWAC induces chemotherapeutic resistance in triple-negative breast cancer by targeting miR-142, upregulating WWP1 and activating the PI3K/AKT pathway. *Molecular cancer* **20**, doi:10.1186/s12943-021-01332-8 (2021).

12.Ni, J., Xi, X., Xiao, S. & Xiao, X. Silencing of circHIPK3 Sensitizes Paclitaxel-Resistant Breast Cancer Cells to Chemotherapy by Regulating HK2 Through Targeting miR-1286. *Cancer management and research* **13,** 5573-5585, doi:10.2147/cmar.S307595 (2021).

13.Ma, J. *et al.* Posttranscriptional regulation of AKT by circular RNA angiomotin-like 1 mediates chemoresistance against paclitaxel in breast cancer cells. *Aging-Us* **11,** 11369-11381, doi:10.18632/aging.102535 (2019).

14.Liu, Y., Dong, Y., Zhao, L., Su, L. & Luo, J. Circular RNA‑MTO1 suppresses breast cancer cell viability and reverses monastrol resistance through regulating the TRAF4/Eg5 axis. *International journal of oncology* **53,** 1752-1762, doi:10.3892/ijo.2018.4485 (2018).

15.Liu, G. *et al.* Circ_0006528 Contributes to Paclitaxel Resistance of Breast Cancer Cells by Regulating miR-1299/CDK8 Axis. *OncoTargets and therapy* **13,** 9497-9511, doi:10.2147/ott.S252886 (2020).

16.Liang, Y. *et al.* circKDM4C suppresses tumor progression and attenuates doxorubicin resistance by regulating miR-548p/PBLD axis in breast cancer. *Oncogene* **38,** 6850-6866, doi:10.1038/s41388-019-0926-z (2019).

17.Li, H., Li, Q. & He, S. Hsa_circ_0025202 suppresses cell tumorigenesis and tamoxifen resistance via miR-197-3p/HIPK3 axis in breast cancer. *World journal of surgical oncology* **19,** 39, doi:10.1186/s12957-021-02149-x (2021).

18.Hao, J., Du, X., Lv, F. & Shi, Q. Knockdown of circ_0006528 Suppresses Cell Proliferation, Migration, Invasion, and Adriamycin Chemoresistance via Regulating the miR-1236-3p/CHD4 Axis in Breast Cancer. *The Journal of surgical research* **260,** 104-115, doi:10.1016/j.jss.2020.10.031 (2021).

19.Xie, H. & Zheng, R. Circ_0085495 knockdown reduces adriamycin resistance in breast cancer through miR-873-5p/integrin β1 axis. *Anti-cancer drugs* **33,** e166-e177, doi:10.1097/cad.0000000000001174 (2022).

20.Wang, Q. *et al.* Hsa_circ_0092276 promotes doxorubicin resistance in breast cancer cells by regulating autophagy via miR-348/ATG7 axis. *Transl Oncol* **14,** 101045, doi:10.1016/j.tranon.2021.101045 (2021).

21.Huang, P. *et al.* A Comprehensive RNA Study to Identify circRNA and miRNA Biomarkers for Docetaxel Resistance in Breast Cancer. *Frontiers in oncology* **11,** 669270, doi:10.3389/fonc.2021.669270 (2021).

22.Yang, S. *et al.* Knockdown circTRIM28 enhances tamoxifen sensitivity via the miR-409-3p/HMGA2 axis in breast cancer. *Reproductive biology and endocrinology : RB&E* **20,** 146, doi:10.1186/s12958-022-01011-3 (2022).

23.Wang, L., Yang, X., Zhou, F., Sun, X. & Li, S. Circular RNA UBAP2 facilitates the cisplatin resistance of triple-negative breast cancer via microRNA-300/anti-silencing function 1B histone chaperone/PI3K/AKT/mTOR axis. *Bioengineered* **13,** 7197-7208, doi:10.1080/21655979.2022.2036894 (2022).

24.Liu, J. *et al.* Circular RNA circMET contributes to tamoxifen resistance of breast cancer cells by targeting miR-204/AHR signaling. *Biochem Biophys Res Commun* **627,** 200-206, doi:10.1016/j.bbrc.2022.07.097 (2022).

25.Ling, Y. *et al.* circCDYL2 promotes trastuzumab resistance via sustaining HER2 downstream signaling in breast cancer. *Molecular cancer* **21,** 8, doi:10.1186/s12943-021-01476-7 (2022).

26.Liang, X., Liu, X., Song, Z., Zhu, J. & Zhang, J. Hsa_circ_0097922 promotes tamoxifen resistance and cell malignant behaviour of breast cancer cells by regulating ACTN4 expression via miR-876-3p. *Clinical and experimental pharmacology & physiology*, doi:10.1111/1440-1681.13702 (2022).

27.Chen, J. *et al.* CircRNA_0044556 diminishes the sensitivity of triple‑negative breast cancer cells to adriamycin by sponging miR‑145 and regulating NRAS. *Molecular medicine reports* **25**, doi:10.3892/mmr.2021.12567 (2022).

28.Yao, Y., Li, X., Cheng, L., Wu, X. & Wu, B. Circular RNA FAT atypical cadherin 1 (circFAT1)/microRNA-525-5p/spindle and kinetochore-associated complex subunit 1 (SKA1) axis regulates oxaliplatin resistance in breast cancer by activating the notch and Wnt signaling pathway. *Bioengineered* **12,** 4032-4043, doi:10.1080/21655979.2021.1951929 (2021).

29.Sang, Y. *et al.* circRNA_0025202 Regulates Tamoxifen Sensitivity and Tumor Progression via Regulating the miR-182-5p/FOXO3a Axis in Breast Cancer. *Molecular therapy : the journal of the American Society of Gene Therapy* **29,** 3525-3527, doi:10.1016/j.ymthe.2021.11.002 (2021).

30.Huang, L., Ma, J. & Cui, M. Circular RNA hsa_circ_0001598 promotes programmed death-ligand-1-mediated immune escape and trastuzumab resistance via sponging miR-1184 in breast cancer cells. *Immunologic research* **69,** 558-567, doi:10.1007/s12026-021-09237-w (2021).
